# EHMTI-0319. TMS-measured cortical excitability do not change by migraine phase: a blinded longitudinal study

**DOI:** 10.1186/1129-2377-15-S1-E38

**Published:** 2014-09-18

**Authors:** M Uglem, PM Omland, T Sand

**Affiliations:** 1 Department of Neuroscience, Norwegian University of Science and Technology, Trondheim, Norway

## Introduction

Many migraineurs have increased sensitivity to light, sounds, odours or sensory stimuli, particularly in the premonitory phase and during the headache attack. These symptoms may be caused by alterations in cortical excitability. Transcranial magnetic stimulation (TMS) measurements of cortical excitability in migraineurs have yielded conflicting results, possibly due to large interindividual differences, different procedures and lack of blinding.

## Aim

Do TMS-measurements of cortical excitability change in relation to a migraine attack?

## Methods

Resting motor threshold (RMT), cortical silent period (CSP) and paired-pulse TMS (ppTMS) were measured on four different days in 16 migraineurs. All participants signed an informed consent form and the Regional Committee for Medical and Health Research Ethics approved the study. Measurements were classified as interictal, preictal or ictal by headache diaries with a one-day cut-off. Ten interictal-preictal and 7 interictal-ictal individual pairs were analysed with paired samples Student’s t-test for RMT and CSP and repeated measures ANOVA with period and inter-stimulus-interval as within-subject factors for ppTMS.

## Results

There were no statistically significant intraindividual changes neither in RMT, CSP nor ppTMS (p > .17).

## Conclusion

Cortical excitability measured by TMS did not change from the period between a migraine attack to right before or during an attack. However, the results should be interpreted with some caution due to the rather small sample size.

No conflict of interest.

